# Transformative Role of Artificial Intelligence in Reporting Haematology Cases: A Case Report

**DOI:** 10.7759/cureus.73274

**Published:** 2024-11-08

**Authors:** Sarandeep S Puri, Ankur K Lath, Neha Goel, Pushkar D Admane, Pradeep Garg, Renu Ethirajan

**Affiliations:** 1 Pathology, GS Medical College and Hospital, Hapur, IND; 2 Pathology, Horiba, New Delhi, IND; 3 Microbiology, GS Medical College and Hospital, Hapur, IND; 4 Pathology, Horiba, Nagpur, IND; 5 Surgery, GS Medical College and Hospital, Hapur, IND; 6 Research and Development, SigTuple Technologies Private Limited, Bangalore, IND

**Keywords:** ai, ai and robotics in healthcare, artificial intelligence in healthcare, haematology, healthcare technology, reporting

## Abstract

Artificial intelligence (AI) is transforming haematology reporting by improving accuracy, standardisation, and speed, addressing the need for timely and precise diagnostics. This study explores the use of the AI100 (SigTuple Technologies Private Limited, Bangalore, India) automated machine, a smart robotic microscope designed to automate the microscopic analysis of peripheral blood smears. Through the analysis of four haematology cases, this study demonstrates how AI technology facilitates efficient cell identification, enhances risk stratification, enables early detection of abnormalities, and accelerates diagnostic turnaround times. These advancements support pathologists in delivering improved patient care by augmenting traditional diagnostic methods. While AI can streamline processes and increase diagnostic accuracy, it is intended to complement, rather than replace, the expertise and judgement of skilled pathologists.

## Introduction

Haematology is a diverse field that involves the diagnosis and treatment of disorders related to red blood cells (RBCs), white blood cells (WBCs), and platelets, including both non-neoplastic and neoplastic conditions [1]. Timely and accurate reporting of haematology cases is crucial, as it directly influences patient care and treatment planning [2,3]. The rapid advancements in technology, particularly in artificial intelligence (AI), have brought transformative changes to diagnostic practices, including haematology. The present study aims to explore the role of AI in haematology reporting, specifically through the use of the AI100 automated machine (SigTuple Technologies Private Limited, Bangalore, India). This AI-driven, smart robotic microscope automates the microscopic review of peripheral blood smears, enhancing diagnostic precision and efficiency. We present four haematology cases diagnosed using this technology, demonstrating the benefits of AI in automating cell identification, improving risk assessment, and facilitating quicker diagnostic decisions. By examining these cases, we highlight how AI can be integrated into traditional pathology practices to improve the quality of patient care.

## Case presentation

**Figure 1:**
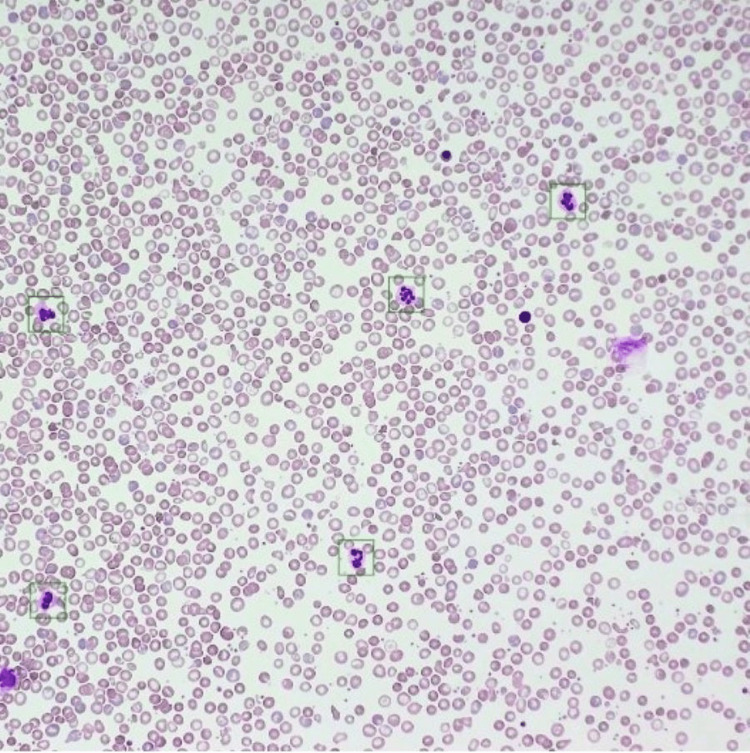
The microphotograph shows the microscopic view of the peripheral blood smear report from SigTuple’s AI100 analyser.

SigTuple’s AI100, in partnership with Horiba India Private Limited (New Delhi, India), has been used to diagnose these four cases. In AI100, a closed-loop controlling mechanism is used for focusing on the cells. This is accomplished by iterative capture of images, evaluating a sharpness measure of the image, commanding a change in Z position (focus axis), and continuing with image analysis and Z movement until the sharpest image is obtained. Another routine, which identifies the most optimal region of the analysis of the specimen, is also implemented to avoid visiting suboptimal or empty regions. Several of the image properties, such as sharpness and contrast, are used as quality metrics for declaring whether the images are sharp. Once images of a specimen from the device are captured, they are forwarded to the cloud-based software for analysis, where all the cells in these images are analysed. The scan begins with capturing an image from the optimal area identified in the previous step. From this image, the stained WBCs are extracted and classified. This process of capturing images continues in an outwardly growing spiral pattern until the required number of images are captured. The number of images required is based on the scan mode selected. Each image is checked for quality indicators to ensure it can be used for further analysis and rejected if not. If enough good-quality images are not available, an appropriate error message is thrown, and the process ends without further analysis (Figure 1) [3-5].

Case one

*A Case of Alpha (*α)*-Thalassaemia Trait*

**Figure 2:**
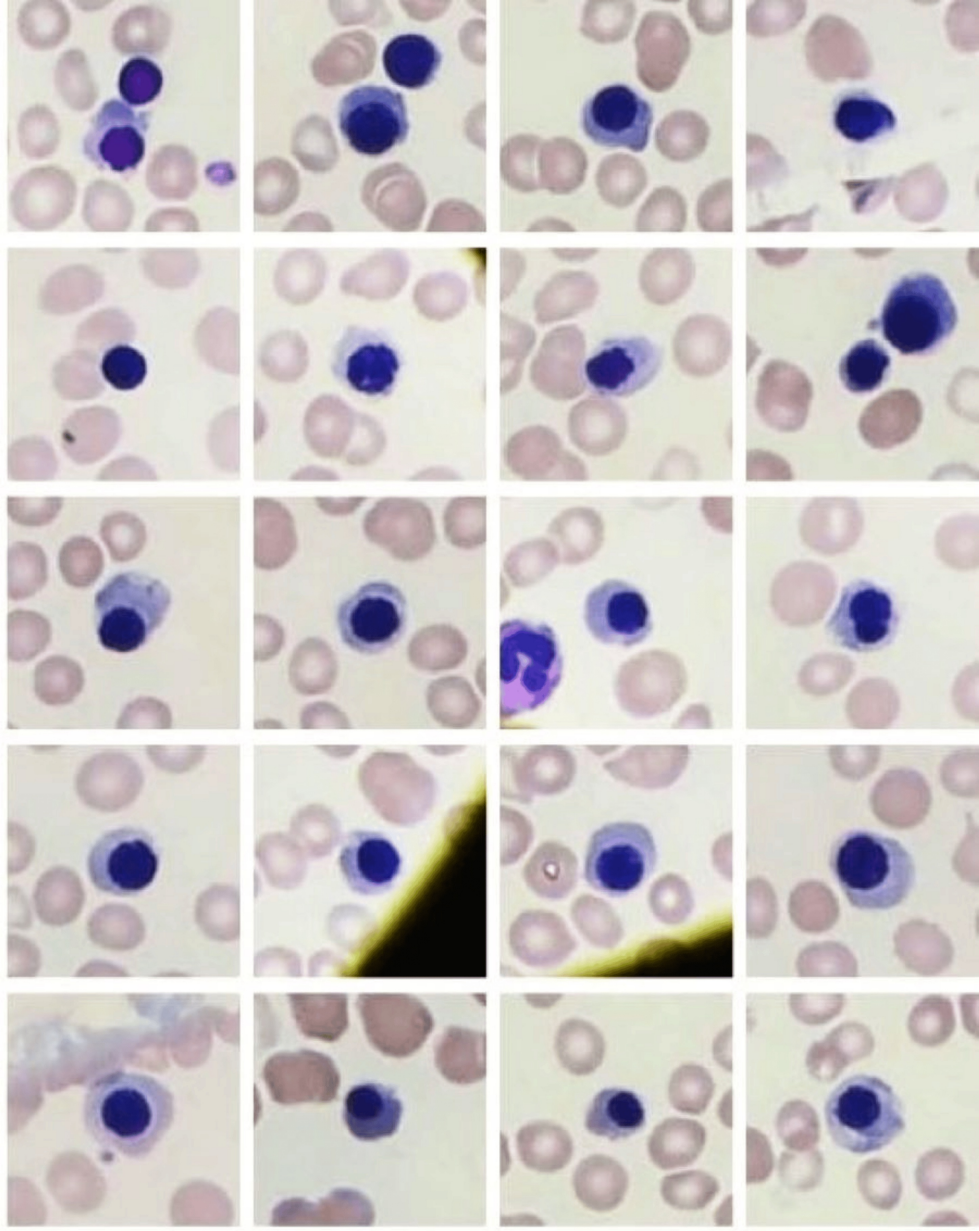
The microphotograph shows nucleated red blood cells in all the panels.

**Figure 3:**
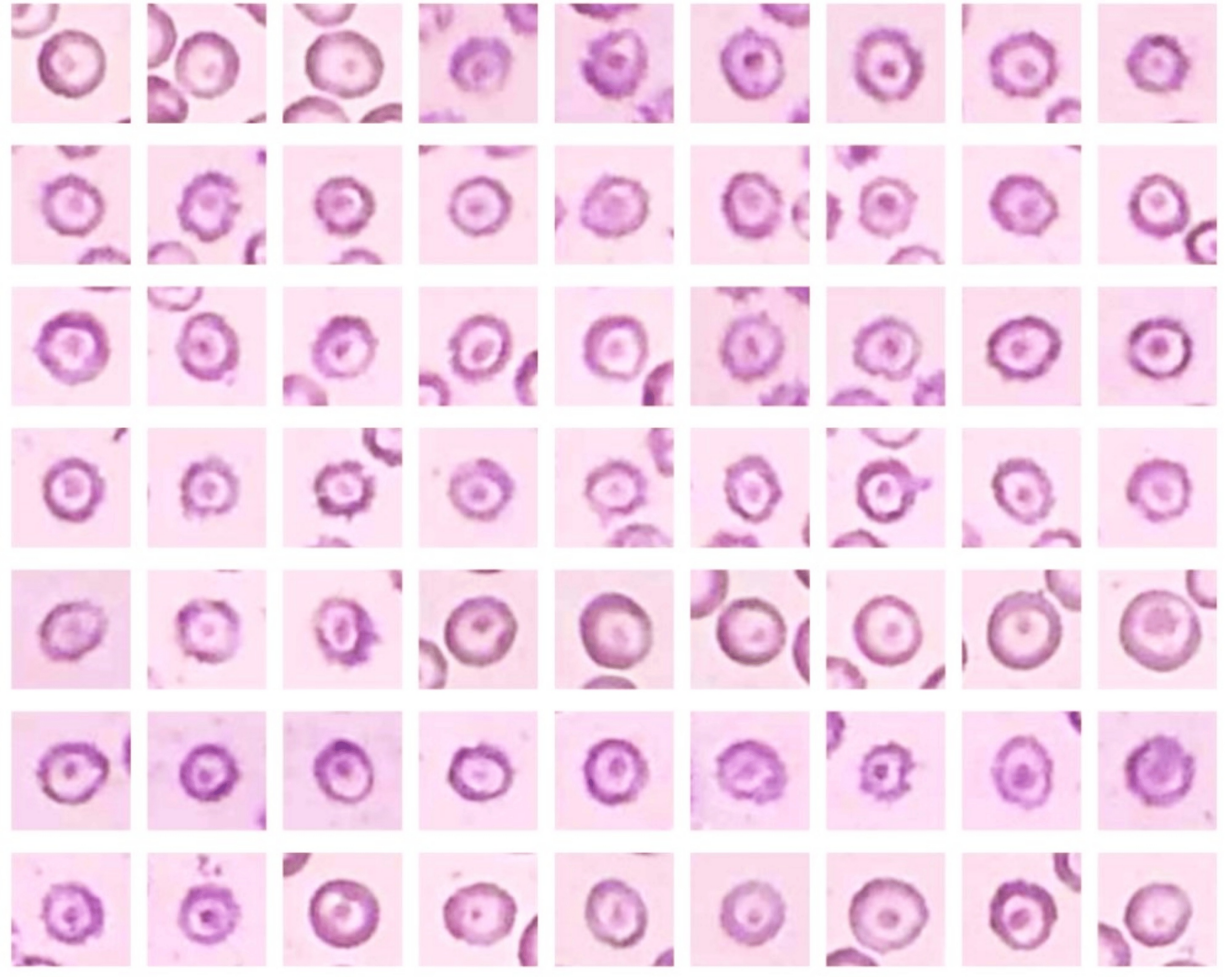
The microphotograph shows target cells in all the panels.

A 26-year-old male visited a tertiary healthcare centre with complaints of persistent low haemoglobin (Hb) in spite of previous iron therapy and mild on-and-off fatigue. On examination, he had pallor but no icterus or hepatosplenomegaly. A relevant anaemia workup was ordered by the clinician. The Hb was 10.6 g/dl, the red cell distribution width (RDW) was 13.1% (coefficient of variation (CV), the WBC count was 8.5 x 103/ul, and the platelet count was 160 x 103/ul. The differential count was within normal limits. Serum iron levels were 150 μg/dl and ferritin was 290 ng/ml. A peripheral blood smear was analysed on Sigtuple AI100, which corroborated the CBC findings and revealed a microcytic hypochromic blood picture with mild to moderate anisopoikilocytosis. Microcytic hypochromic RBCs, elliptocytes, target cells, and occasional nucleated red blood cells (NRBCs) were captured and classified by this AI-equipped digital morphology analyser. Along with the visual evidence of captured fields of vision (FOVs), these findings were presented to the pathologist for review and final opinion. As the patient had a trial of iron therapy and his iron and ferritin levels were normal, the clinician ordered Hb electrophoresis to evaluate further, which turned out to be normal with haemoglobin A (HbA) of 97%, haemoglobin A2 (HbA2) of 2.5%, and haemoglobin F (HbF) of 0.5%. So, the pathologist shared these results with a hematopathologist based out in a distant city through the digital pathology platform and took a second opinion. The haematologist reviewed the images of the peripheral blood smear (PBS) through AI100 and the other reports and strongly recommended genetic testing of the patient’s Hb status to rule out α-thalassaemia. The results showed the presence of only 2α genes with deletion of the remaining 2α genes, and the diagnosis of α- thalassaemia was clinched. The patient was counselled accordingly, and the importance of premarital genetic counselling for him and his siblings to avoid the possibility of birth of any child with haemoglobin H (HbH) disease or Hb Bart’s and the severity of these disease entities was conveyed to the patient (Figures 2,3).

Case two

A Case of Acute Myeloid Leukemia (AML)

A case of AML with cuplike blasts was reported through remote access on the AI100 analyzer. A pathologist who was travelling abroad to attend a conference had to report an emergency sample of a case of anaemia with leukocytosis and severe thrombocytopenia. The CBC findings were: haemoglobin: 7.8 g/dl; WBC count: 161.4 x 10^9 cells/L; platelet count: 27 x 10^3 cells/ul. The automated cell counter gave WBC differential counts of neutrophils (0.5%), lymphocytes (19.3%), monocytes (80%), eosinophils (0.1%), and basophils (0.1%). The doctor chose to report the peripheral blood smear remotely on SigTuple AI100, as he was confident of the visual evidence that this AI-equipped digital morphology analyser provides. The digital images of the peripheral blood smear, along with the RBC grading (size and shape-based), WBC differential count, and platelet count, were displayed to him on the Mandara, AI100's remote reporting platform. AI100 had flagged 86% of the WBCs as atypical cells/blasts and highlighted severe thrombocytopenia. He reviewed these images remotely and immediately approved the report as acute leukaemia.

**Figure 4:**
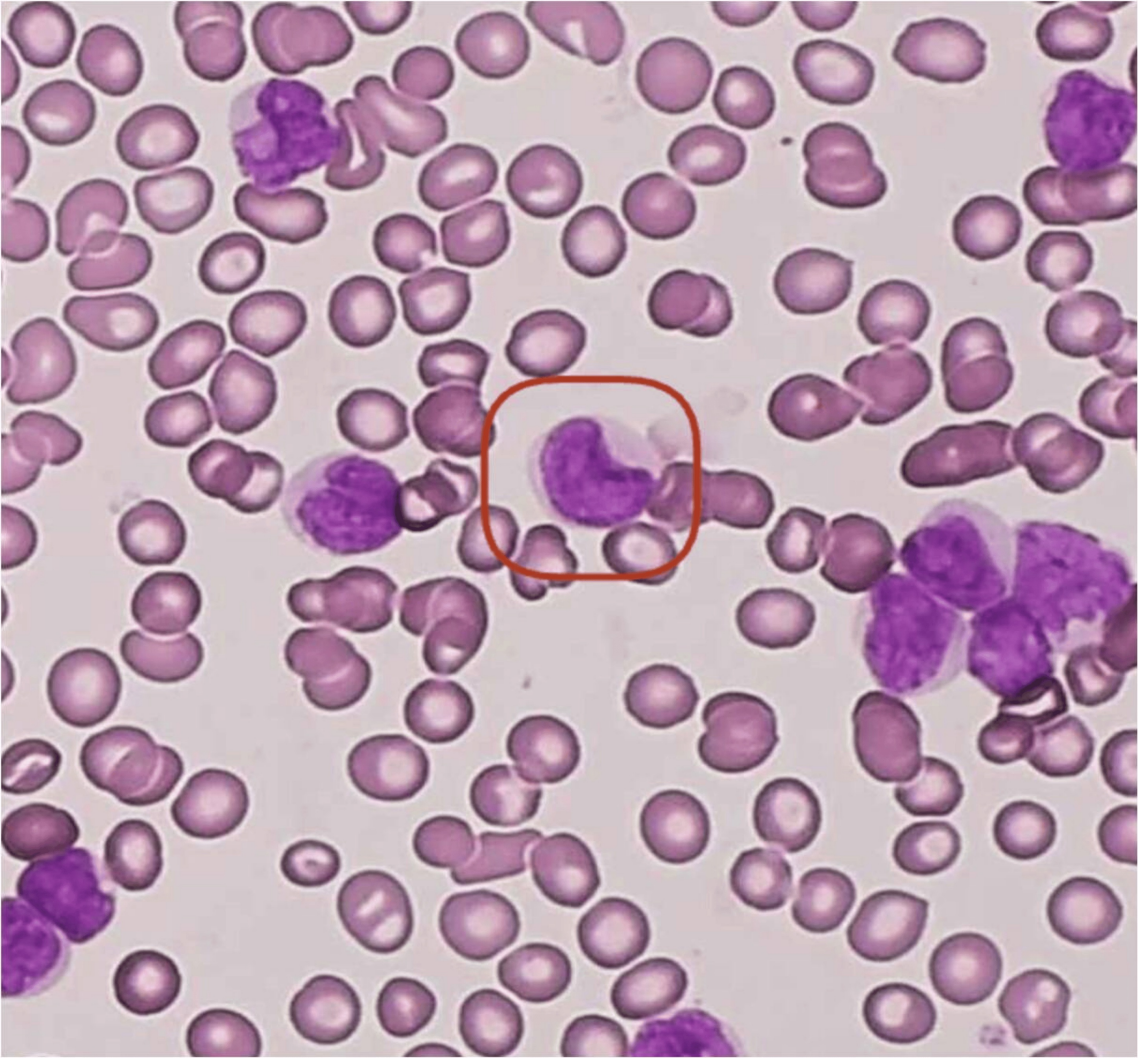
The microphotograph shows cup-like myeloblasts.

**Figure 5:**
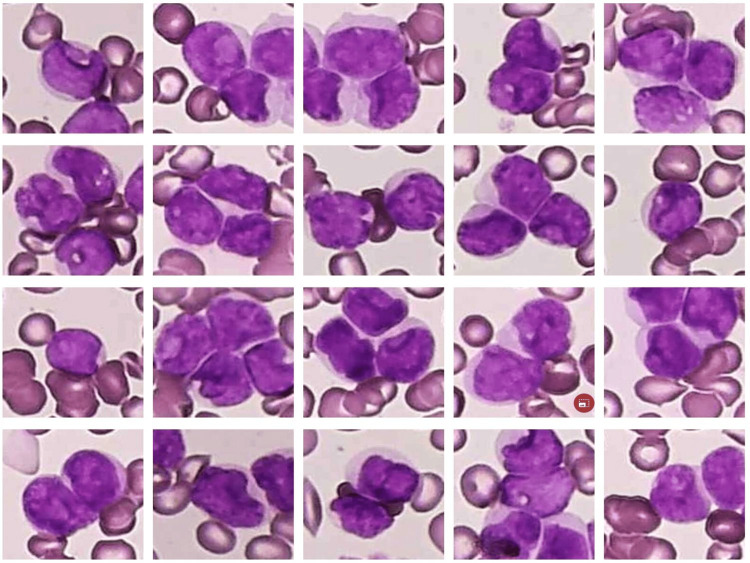
The microphotograph shows atypical cells/blasts.

He also observed that more than 10% of the blasts had “cuplike nuclear invagination” (spanning more than 25% of the nuclear area). Cases of AML with this distinctive morphology have been known to be associated with a high frequency of NPM1 and FLT3-ITD mutations. In the past few years, the poor prognostic impact of FLT3-ITD and the favourable prognosis associated with NPM1 genes, if not associated with any other mutations, have been recognised. The molecular biology, the therapeutic targets, and the prognosis are different in these cases. Hence, the pathologist signed out the PBS report, advising further molecular testing for NPM1 and FLT3 mutations, which are crucial in the clinical management of this case. Thus, morphological assessment plays a crucial role in prioritising the molecular workup in such AML cases and helps the pathologist in offering an early, in-depth diagnosis and the clinician in streamlining informed clinical management. This case is a perfect example to prove that AI100 can and is revolutionising the present clinical practice for better patient care (Figures 4,5). 

Case Three

A Case of Eosinophilia

**Figure 6:**
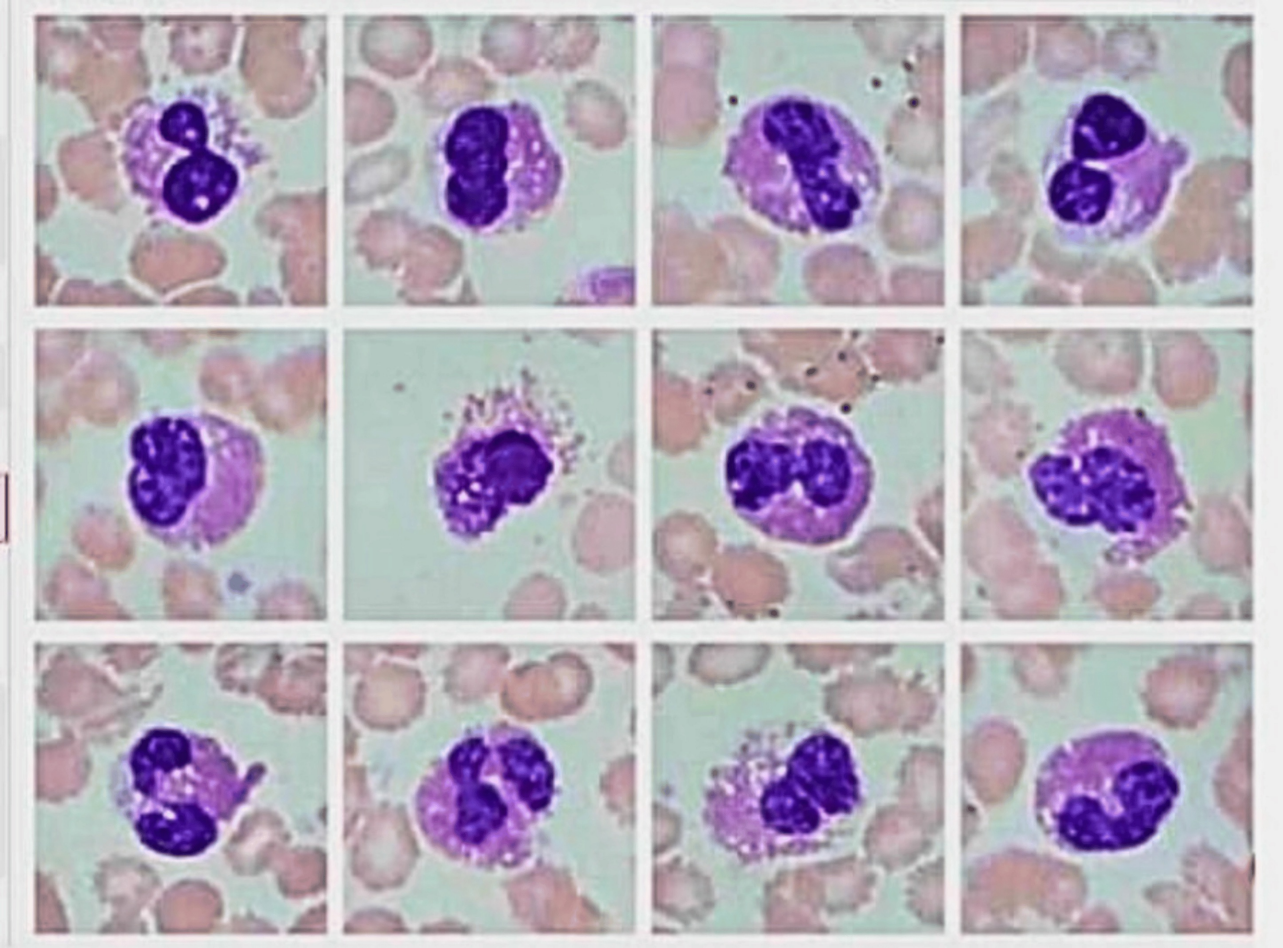
The microphotograph shows eosinophilia

A 40-year-old female patient came for a routine health check-up with an unremarkable previous medical history. Routine screening tests such as CBC and PBS were done. The PBS was scanned in SigTuple AI100, an AI-enabled device, which identified and categorised WBC, RBC, and platelets. Based on the AI100 results, the pathologist confirmed eosinophilia. The AI-enabled digital image analysers automate the blood smear analysis and enable faster slide reviews. AI100 analyses the peripheral blood smear and performs the WBC differential counts much faster than manual microscopy, producing high-resolution images. It has a high accuracy rate in the detection of eosinophils (Table 1, Figure 6).

**Table 1:** The patient's white blood cell (WBC) absolute counts

Name	Count	Percentage
Neutrophils	137	23.62%
Band neutrophils	1	0.17%
Hypersegmented*		
Lymphocytes	101	17.41%
Reactive lymphocytes*		
Monocytes	40	6.89%
Eosinophils	293	50.51%
Basophils	3	0.51%
Immunoglobulin (IG)	6	1.03%
Promyelocytes	3	0.51%
Myelocytes	3	0.51%
Metamyelocytes	3	0.51%
Atypical/blast	0	0%

Case four

A Case of Chronic Lymphoproliferative Disorder

**Figure 7:**
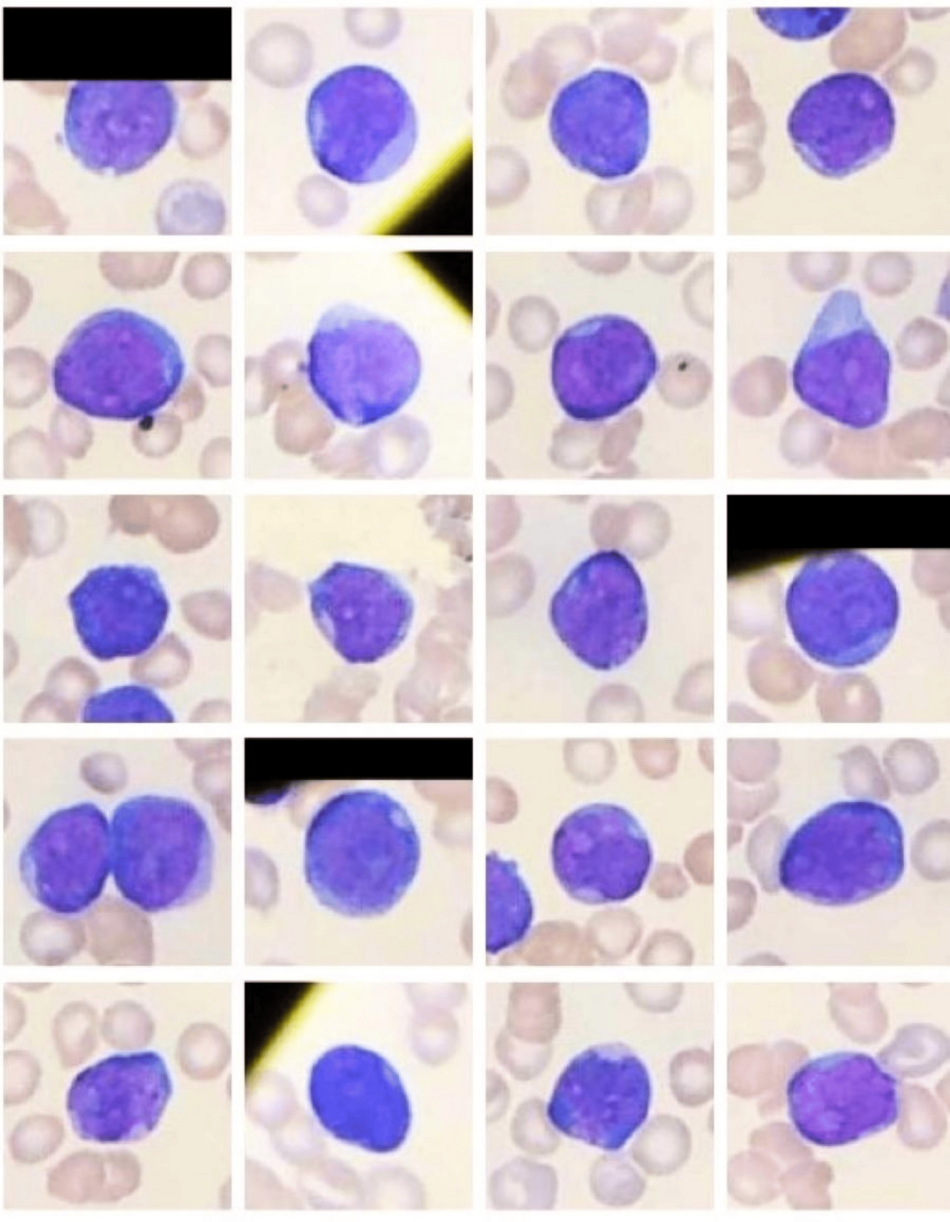
The microphotograph shows small, mature B cells.

A case was reported using the AI100 analyser. Here the results of AI100 showed visual evidence of the monomorphic small mature B cells and the typical basket cells/smudge cells found in the peripheral smears, which made the diagnosis by remote access easy, comfortable, and decisive for the pathologist (Table 2, Figure 7).

**Table 2:** The patient's white blood cell (WBC) absolute count

Name	Count	Percentage
Neutrophils	321	22.06%
Band neutrophils	-	-
Hypersegmented*	-	-
Lymphocytes	1071	73.61%
Reactive lymphocytes*	-	-
Monocytes	48	3.3%
Eosinophils	1	0.07%
Basophils	2	0.14%
Immunoglobulin (IG)	3	0.2%
Promyelocytes	-	-
Myelocytes	1	0.06%
Metamyelocytes	2	0.13%
Atypical/Blast	9	0.61%
Atypical lymphocytes*	-	-

## Discussion

This study demonstrates that the integration of AI in haematology reporting, through the use of the AI100 automated machine, enhances diagnostic efficiency and accuracy. The AI-driven machine successfully automated cell identification, improved risk stratification, facilitated early detection of abnormalities, and significantly reduced turnaround times across four haematology cases [4,5]. These findings suggest that AI technology can optimise workflow while maintaining high diagnostic precision, which is critical in improving patient care. The results of this study align with the growing body of research emphasising the potential of AI in transforming diagnostic practices. Previous studies have highlighted AI’s ability to standardise complex processes and reduce human error, particularly in areas such as cell morphology and classification [5-7]. Our findings corroborate this, showing that AI can complement pathologists by handling routine tasks, allowing them to focus on more complex cases. However, AI is not without limitations. While it reduces manual workload and errors, the technology still relies on human oversight, particularly in cases where clinical judgment is required [3].

Comparison with previous studies

The present study, using the AI100 automated machine, demonstrated significant improvements in diagnostic accuracy, speed, and standardisation across four haematological cases (alpha-thalassaemia trait, AML, eosinophilia, chronic lymphoproliferative disorder). It showcased AI’s ability to accurately identify cell types, enable remote access, and provide faster reporting while complementing human expertise. Compared to previous studies, such as El Alaoui et al., which highlighted AI’s potential but emphasised the need for extensive training data [1], and Acharya et al., which noted the effectiveness of AI in AML detection but faced workflow integration challenges, the present study offered more practical case examples [2]. It also aligned with findings from Chari and Prasad, which pointed out the need for high-quality images [3], and Fan et al., which discussed the promise of AI in automating blood film analysis but stressed robust data validation [5]. The current study underscores AI's impact on diagnostics while emphasising the need for human oversight.

Implications for clinical practice and research

The implications of these findings are significant for both clinical practice and future research. In clinical settings, AI can be implemented as a tool to improve efficiency and reduce diagnostic turnaround times, particularly in high-volume laboratories where timely reporting is crucial [8]. By automating routine tasks, AI can help pathologists prioritise more critical or complex cases, ultimately enhancing patient outcomes [9]. However, AI systems must be viewed as supplementary tools that work in tandem with human expertise rather than replacements for it [10]. For research, future studies should focus on improving the robustness of AI algorithms by incorporating more diverse and comprehensive datasets [11]. This will ensure that AI models are capable of handling a broader spectrum of cases, including rare and complex disorders. Additionally, more research is needed to evaluate the long-term impact of AI integration on diagnostic accuracy and workflow efficiency in clinical settings [12].

This study contributes to the current understanding of AI’s role in haematology reporting by demonstrating its potential to enhance diagnostic accuracy and workflow efficiency [13]. While AI-driven tools offer significant advantages in terms of speed and standardisation, they should be integrated thoughtfully, with recognition of their limitations. The future of haematology reporting will likely involve a balanced approach where AI supports pathologists, enabling them to make quicker and more informed decisions while maintaining clinical judgement at the forefront [14].

## Conclusions

This study highlights the transformative role of AI in haematology reporting, with the AI100 automated machine demonstrating its ability to improve diagnostic efficiency and accuracy. Key advancements include the automation of cell identification, risk stratification, and early abnormality detection, which contribute to faster turnaround times and enhanced patient care. Although AI offers significant benefits in standardising and expediting the diagnostic process, its integration should be viewed as complementary to human expertise. Artificial intelligence supports pathologists by reducing errors and optimising workflows, but it cannot replace the nuanced judgement required in complex clinical cases. The future of haematology reporting lies in the balanced integration of AI-driven tools with the clinical knowledge and experience of healthcare professionals, ensuring improved outcomes for patients.
